# Case report: Distinctive cardiac features and phenotypic characteristics of Noonan syndrome with multiple lentigines among three generations in one family

**DOI:** 10.3389/fcvm.2023.1225667

**Published:** 2023-08-24

**Authors:** Chon-Hou Chan, Man-Fong Chu, U-Po Lam, Toi-Meng Mok, Weng-Chio Tam, Brian Tomlinson, Ricardo Coelho, Màrio Évora

**Affiliations:** ^1^ Department of Dermatology, Centro Hospitalar Conde São Januário, Macao, Macao SAR, China; ^2^ Department of Cardiology, Centro Hospitalar Conde São Januário, Macao, Macao SAR, China; ^3^ Faculty of Medicine, Macau University of Science and Technology, Taipa, Macao SAR, China

**Keywords:** hypertrophic cardiomyopathy, Noonan syndrome with multiple lentigines, inherited disorder, sudden cardiac death, echocardiography

## Abstract

Noonan syndrome with multiple lentigines (NSML, formerly known as LEOPARD syndrome) is a variant of Noonan syndrome which is an autosomal dominant disorder. Most cases of NSML are secondary to mutations of the protein-tyrosine phosphatase nonreceptor type 11 (*PTPN11*). Hypertrophic cardiomyopathy (HCM) remains the most frequent and serious cardiac abnormality in this inherited syndrome, and it may lead to sudden cardiac death related to HCM-associated outflow obstruction and fatal arrhythmia. Beyond cardiac involvement, NSML may present with multiple lentigines, ocular hypertelorism, genital anomalies, short stature and deafness. Herein, we report three patients with NSML among three generations in one family, all presenting with multiple lentigines, HCM and other distinctive clinical and molecular features, including facial dysmorphism, deafness, family history of sudden death and *PTPN11* mutations. This case series highlights the importance of early echocardiography examinations for patients with NSML. Careful family screening and genetic counselling are also necessary, especially in patients with diffuse lentigines or a history of sudden death among family members. We also discuss the distinctive cardiac features and phenotypic characteristics at different stages of NSML, including childhood, adulthood and elderhood.

## Introduction

Noonan syndrome with multiple lentigines (NSML), formerly known as LEOPARD syndrome, is a rare multi-systemic disorder that is inherited in an autosomal-dominant manner. It was first described by **Gorlin et al.** in 1969, and is characterized by seven major features: lentigines, electrocardiographic conduction abnormalities, ocular hypertelorism, pulmonary stenosis, abnormalities of genitalia, growth retardation, and deafness (1). About 200 cases have been reported in the English literature to date, but the incidence of NSML is not well documented. NSML is a “RASopathy” related to gene variants within the Ras/Mitogen-activated protein kinase (Ras/MAPK) pathway (2). Approximately 85% of patients with NSML are associated with missense mutations in exon 7, 12 or 13 of the *PTPN11* on chromosome 12q24 (3, 4). *PTPN11* mutations down-regulate the activity of Src homology-2 domain-containing protein-tyrosine phosphatase 2 (SHP-2), which enhances melanin synthesis in melanocytes causing diffuse lentigines (5). Hypertrophic cardiomyopathy (HCM), one of the diagnostic criteria of NSML, can be asymptomatic and easily missed at its early stage. Herein, we report three Han Chinese patients with NSML among three generations in one family, with an emphasis on their echocardiographic features and importance of family screening.

## Cases presentation

### Case 1 (proband, individual II-3)

**Figure 1:**
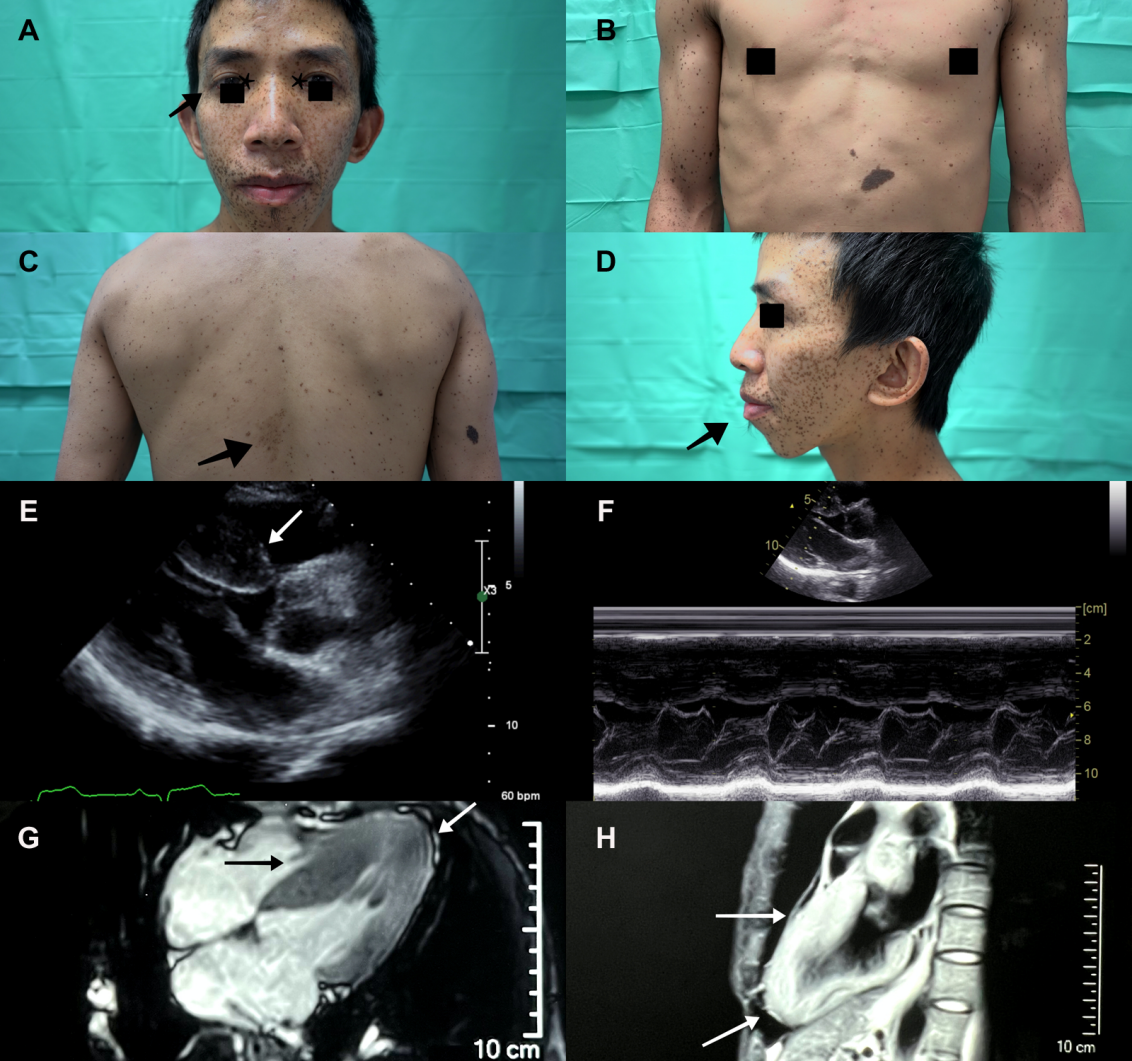
A Diffuse black to brownish lentigines seen mainly on the face of the proband (individual II-3). Ocular hypertelorism (asterisk), ptosis (arrowhead) and thick lips were remarkable. (**B**) Multiple lentigines over the anterior trunk and limbs varied in shape and size, and with diameters ranging from to 5 mm. (**C**) A café au lait macule (arrowhead) was seen on his mid-back. (**D**) Low set ears and retrognathism (arrowhead) were revealed. Echocardiography demonstrated (**E**) eccentric left ventricular hypertrophy (septal wall thickness: 22 mm, arrowhead), (**F**) without left ventricle outflow tract obstruction. (**G,H**) Cardiac magnetic resonance imaging showed hypertrophic segments over septal and apical walls (arrowhead).

**Figure 2:**
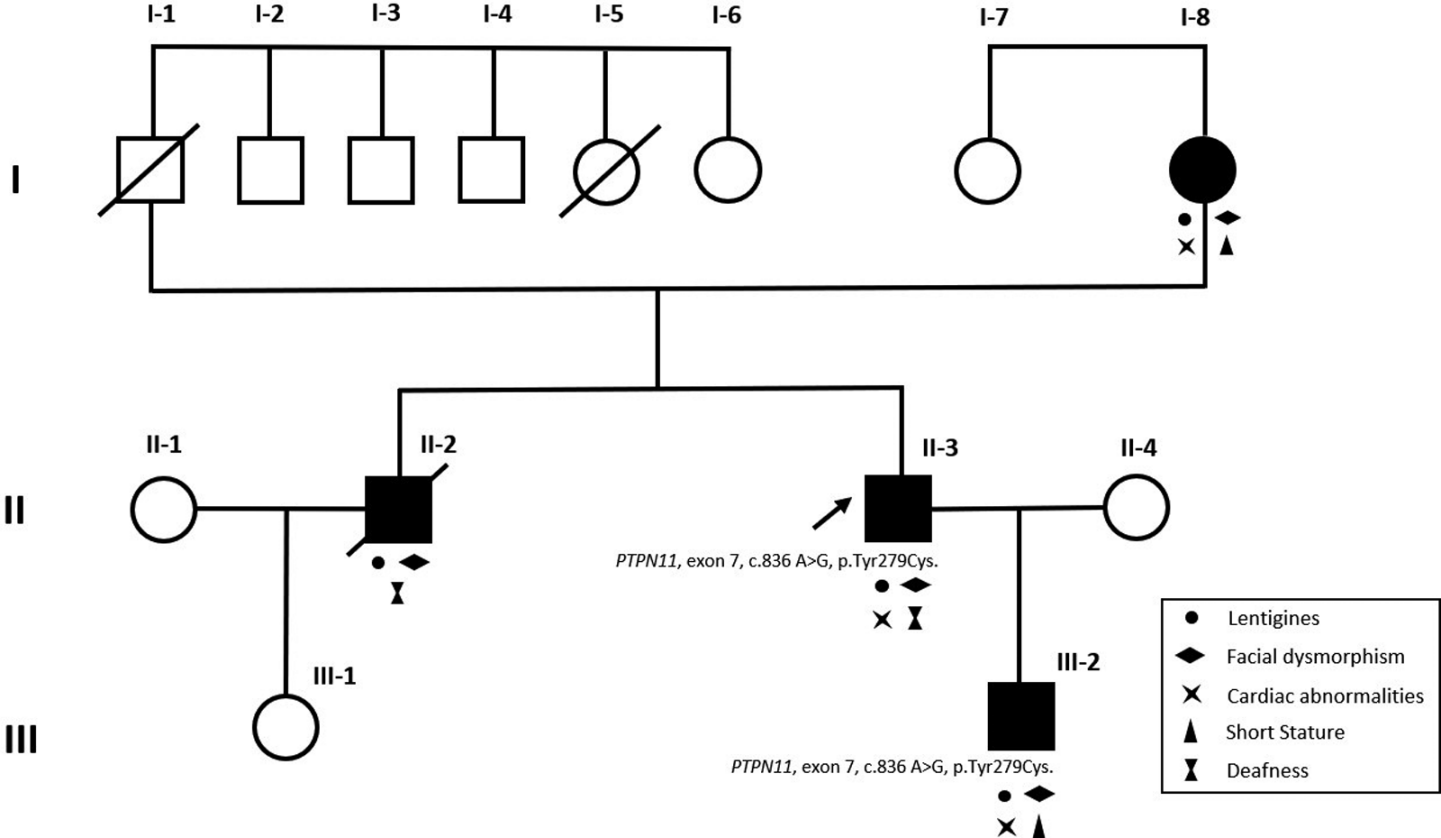
Pedigree of the proband (II-3, arrowhead). The black-filled squares and circle indicated the individuals with clinical evidence of NSML. Both proband (II-3), proband's mother (I-8), elder brother (II-2) and son (III-2) presented with multiple lentigines and facial dysmorphism. Clinical features of NSML were labelled with relevant symbols in the pedigree. Same heterozygous missense mutation of *PTPN11 was* confirmed in proband (II-3) and proband's son (III-2). Two unaffected family members (I-7 and III-1) showed no clinical evidence of NSML. Individual II-2 died at the age of 32 due to sudden cardiac death.

A 36-year-old male with a past history of bilateral congenital hearing loss presented to the dermatology clinic with diffuse black to brownish pigmented macules locating on his face, trunk and four limbs since early childhood, varying in shape and size, and with diameters ranging from 1 to 5 mm (Figures 1A–C). These skin lesions increased in number and darkened during his growing up. Café-au-lait macule was revealed (Figure 1C). Facial dysmorphic features including ocular hypertelorism, ptosis, thick lips (Figure 1A), retrognathism and bilateral low set ears (Figure 1D) were remarkable. He was referred to cardiology for cardiac assessment. The electrocardiogram showed sinus rhythm and left ventricular hypertrophy with strain pattern. Echocardiography demonstrated eccentric left ventricular hypertrophy (septal wall thickness: 22 mm) without outflow tract obstruction (Figures 1E,F, Supplementary Video S1). Cardiac magnetic resonance imaging revealed consistent findings with echocardiography (Figures 1G,H). NSML was confirmed based on the diagnostic criteria proposed by Voron et al. (6). Next generation sequencing revealed a heterozygous missense mutation in the *PTPN11*, exon 7, c.836 A > G, p.Tyr279Cys. This is a well-known genetic variant which has been published previously (3, 4, 7). After assessing his risk of sudden cardiac death (SCD), an implantable cardioverter defibrillator was not strongly indicated for primary prevention. We initiated beta-blockers and suggested him to avoid vigorous physical exercise. Detailed family screening was then performed, which found that his son (individual III-2), elder brother (individual II-2) and mother (individual I-8) also had multiple lentigines since childhood, and that his elder brother (individual II-2) had died at the age of 32 due to SCD (Pedigree, Figure 2).

### Case 2 (proband's son, individual III-2)

**Figure 3:**
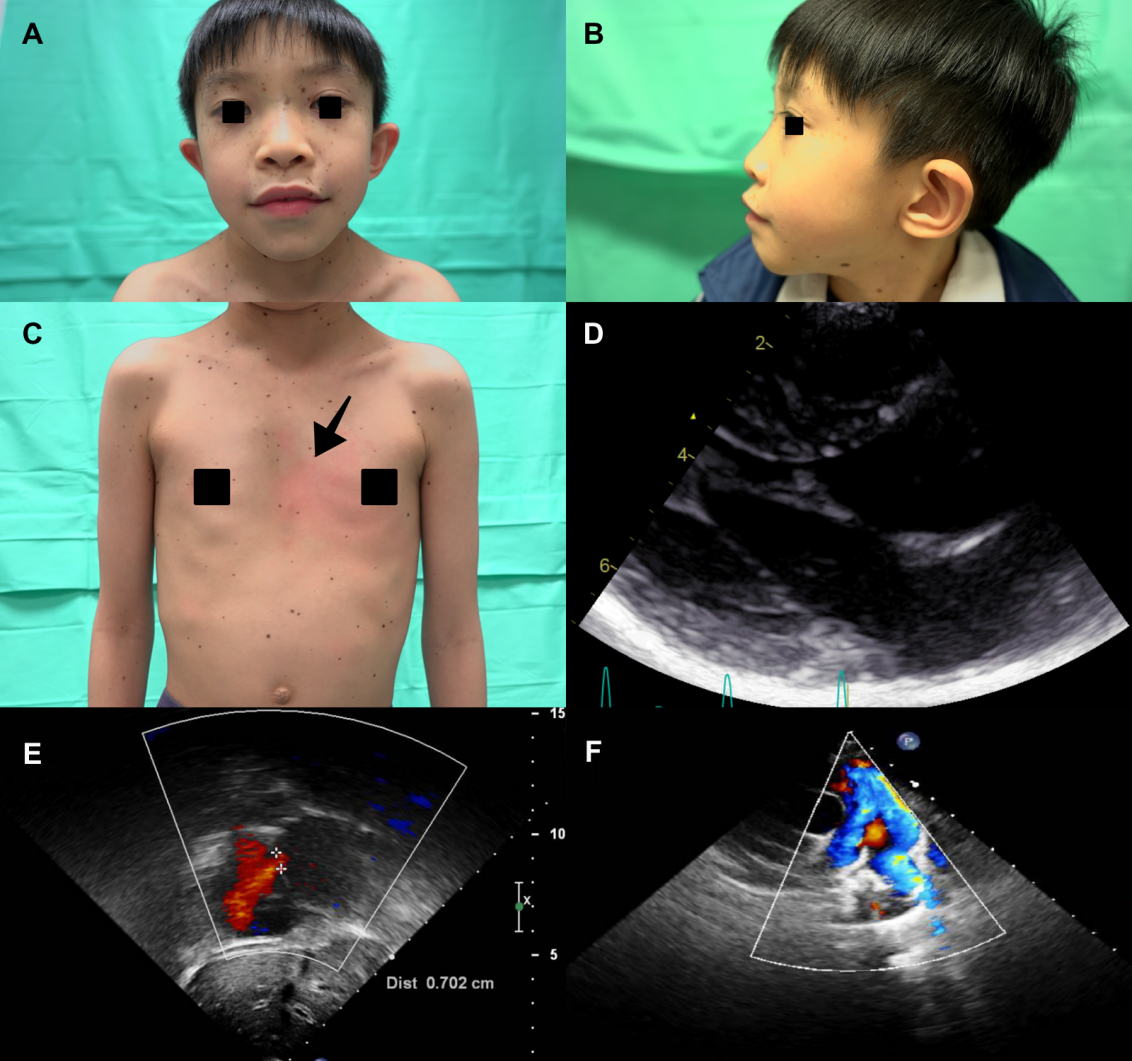
(**A**) The proband's son (individual III-2) presented with diffuse lentigines over the face, thick lips and ocular hypertelorism. (**B**) Low set ears and (**C**) Diffuse lentigines over the trunk and pectus excavatum (arrowhead) were also revealed. Echocardiography showed (**D**) septal wall hypertrophy without intra-cavity or left ventricle outflow tract obstruction. (**E**) Secundum atrial septal defect (cross-mark) (defect size about 7 mm, shunting from left to right). (**F**) Narrowing color-flow at the right pulmonary artery, indicating right pulmonary artery stenosis.

A 8-year-old boy with a 2-year history of multiple lentigines (Figures 3A,C) and two café-au-lait macules on his limbs since birth was found to have short stature (−3.0 standard deviation score from average), ocular hypertelorism (Figure 3A), low set ears (Figure 3B), pectus excavatum (Figure 3C) and systolic murmur upon physical examination. He denied any hearing loss. Echocardiography demonstrated normal left/right ventricle size and function, septal wall hypertrophy (maximal wall thickness: 17 mm) without left ventricle outflow tract obstruction (LVOTO) (Figure 3D and Supplementary Video S2), a secundum atrial septal defect (7 mm, shunting from left to right) (Figure 3E), and right pulmonary artery stenosis (peak pressure gradient around 14 mmHg) (Figure 3F). Next generation sequencing revealed the same heterozygous mutation in the *PTPN11*, exon 7, c.836 A > G, p.Tyr279Cys. He was treated with beta-blockers and serial follow-up at our cardiology department with periodic echocardiography.

### Case 3 (proband's mother, individual I-8)

**Figure 4:**
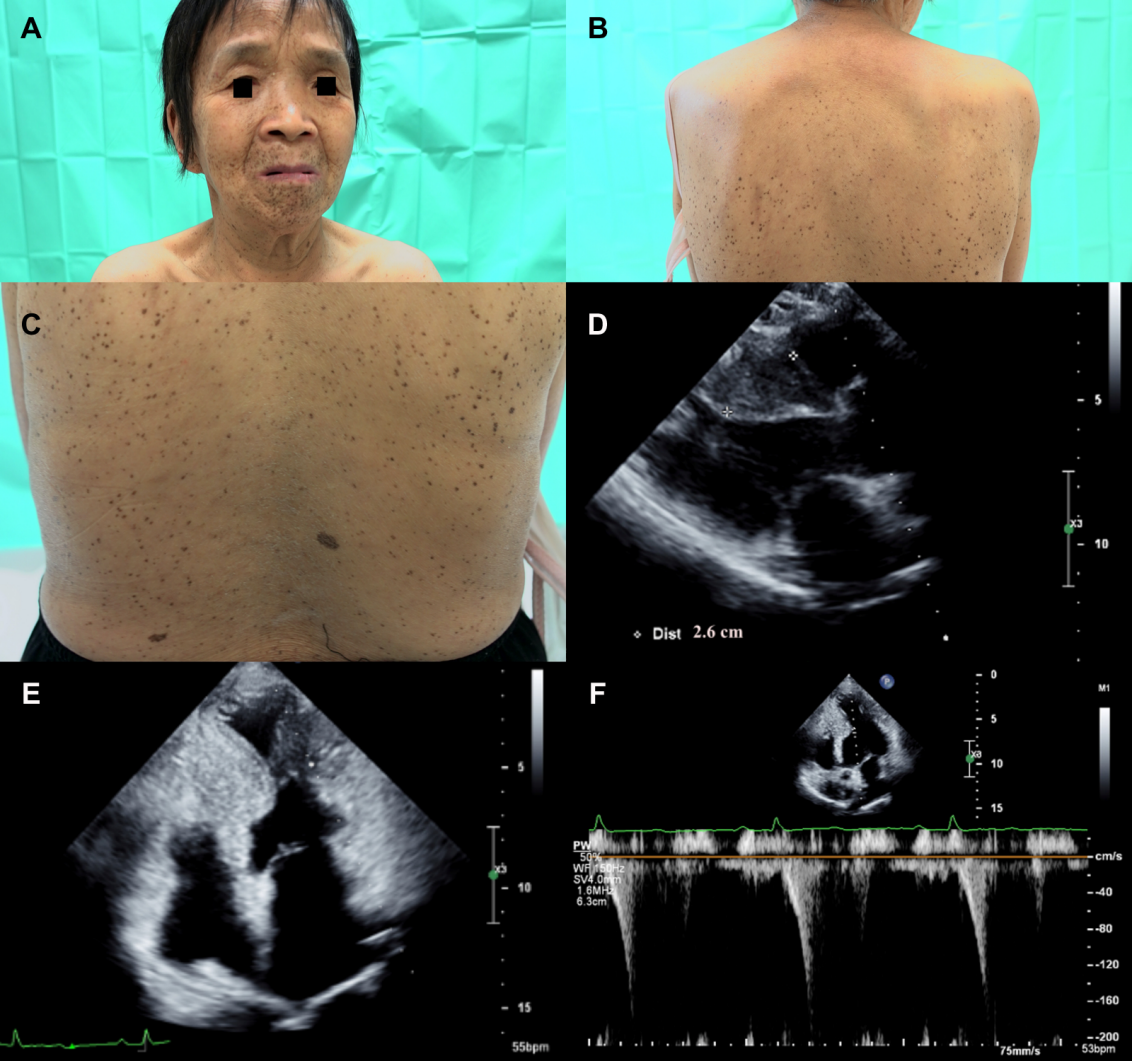
(**A**) The proband's mother (individual I-8) presented with ocular hypertelorism and diffuse black to brownish facial lentigines. (**B**) Numerous lentigines on her back, varying in size and shape. (**C**) Close-up of back lentigines with diameters ranging from 1 mm to 5 mm. Echocardiography revealed (**D**) significant septal wall hypertrophy (septal wall thickness: 26 mm, cross-mark). (**E**) Apical aneurysm formation was revealed with midventricular obstruction. (**F**) Pulsed wave Doppler showed mid-cavity pressure gradient was around 17 mmHg.

A 66-year-old woman with a history of diffuse lentigines since childhood (Figures 4A–C) came to the cardiology clinic with intermittent exertional dyspnea and exercise intolerance in the recent few months. Ocular hypertelorism was revealed (Figure 4A). She had no hearing loss. Electrocardiography showed sinus rhythm with left ventricular hypertrophy using voltage criteria. Echocardiography revealed eccentric septal hypertrophy (septal wall thickness: 26 mm) (Figure 4D), apical aneurysm and mid-cavity obstruction with pressure gradient (17 mmHg) (Figures 4E,F and Supplementary Video S3). Implantation of an implantable cardioverter defibrillator followed by septal wall myomectomy was proposed based on SCD risk stratification (8). Table 1 summarizes the clinical manifestations and cardiac phenotypes of these three patients (individuals II-3, III-2, I-8). Supplementary Table S1 also summarizes clinical phenotypic features of the unaffected family members (individuals I-7 & III-1).

**Table 1:** Clinical characteristics and cardiac phenotypes of the three patients with NSML.

	Proband (individual II-3)	Proband's son (individual III-2)	Proband's mother (individual I-8)
Age of diagnosis	36 y/o	8 y/o	66 y/o
Sex	M	M	F
Lentigines	**+**	**+**	**+**
Café au lait macules	**+**	**+**	**−**
Deafness	**+**	**−**	**−**
Ocular hypertelorism	**+**	**+**	**+**
Genital anomalies	**−**	**−**	**−**
Short stature	**−**	+	+
Ptosis	+	**−**	**−**
Thick lips	+	+	**−**
Low-set ear	+	+	+
Pectus deformity (excavatum/carinatum)	**−**	+	**−**
Intellectual disability	**−**	**−**	**−**
Cryptorchidism	**−**	**−**	n/a
Hypospadias	**−**	**−**	n/a
ECG manifestation	Sinus rhythm, T wave inversion in V1-6, LVH with strain pattern	Sinus rhythm, nonspecific ST-T alternation	Sinus rhythm, biphasic ST-T alternation in anterior leads, LVH
Atrial/ventricular arrhythmia, conduction block	**−**	**−**	**−**
Echocardiography findings	HCM without LVOTO	HCM without LVOTO, Atrial septal defect, pulmonary stenosis	HCM with midventricular obstruction
Left ventricle septal wall thickness	22 mm	17 mm	26 mm
PTPN11 mutation	exon 7, c.836 > G, pc Tyr279Cys (3, 4, 7)	exon 7, c.836 > G, pc Tyr279Cys (3, 4, 7)	Not performed
Indicated for ICD implantation based on SCD risk stratification	**−**	**−**	**+**

HCM, hypertrophic cardiomyopathy; LVH, left ventricle hypertrophy; LVOTO, left ventricle outflow tract obstruction; ICD, implantable cardioverter defibrillator; SCD, sudden cardiac death.

## Discussion

In this case report, we document the clinical manifestations of NSML among three different generations in one family, with an emphasis on the cardiac and cutaneous presentations at different stages of the same disease in childhood, adulthood and elderhood. Of note, none of the three patients received any treatment or evaluations until the diagnosis of NSML was confirmed. Our report demonstrates that NSML-related HCM without treatment can cause left ventricle outflow/midventricular obstruction, leading to sudden cardiac death. We also identified the distinctive clinical manifestations and evolution of the disease, especially the cardiac phenotypes at different stages.

Approximately 95% of cases of NSML are caused by mutations in 3 loci: *PTPN11* on chromosome 12q24, *RAF1* on chromosome 3p25.2, and *BRAF* on chromosome 7q34. About 85% of all cases of NSML are related to *PTPN11* mutations, which are mostly detected on exons 7, 12, and 13 (2). The cardiac phenotypes may be related to specific exon mutations. For example, mutations on exon 7 and 12 are associated with HCM, while mutations on exon 13 are associated with SCD (9, 10). Regarding multiple lentigines formation, plexin B1 can be associated with c-MET, which inhibits the HGF/c-MET pathway by blocking SHP-2 activity, followed by the abrogation of MAPK/ERK and PI3K/AKT activation in melanocytes. This may explain why mutations in SHP-2 cause generalized lentigines (11). Also, the lentigines usually appear first in one's early childhood with main involvement of face and trunk, and then gradually increase in size and numbers with darkening as one grows up. We demonstrated these findings in our case report. The proband's son (individual III-2) presented with fewer lentigines while proband (individual II-3) and proband's mother (individual I-8) were evident with numerous and increased size of lentigines, predominantly distributed on the face and upper part of the trunk.

The *PTPN11*, located in chromosome 12q24, encodes a protein with the Src homology-2 (SH2) domain and tyrosine phosphatase domain containing the active site (12). NS and NSML are two disorders that are categorized as RASopathies. Pathogenic SHP-2 variants are found in NS, NSML and other RASopathies, and large variations among phenotypes have also been observed (13, 14). Several *PTPN11* missense mutations that cause NSML have been identiﬁed and p.Tyr279Cys in exon 7 is one of the most frequent mutations reported in the literature including in one Han Chinese subject (4, 7). Germline mutations in SHP-2 are known to cause both NS and NSML, two clinically similar autosomal dominant developmental disorders (15). Although NS and NSML patients display multiple overlapping phenotypic traits in early childhood, including short stature and facial dysmorphic features, NSML is difficult to differentiate from NS based on clinical manifestation at the early stage of the disease until the presentation of typical multiple lentigines and genetic confirmation. However, some specific features reflect the unique mechanisms of NS and NSML. NS and NSML SHP-2 variants cause opposite effects on the phosphatase activity of SHP-2 (15). Recent studies demonstrated that NS was associated with gain-of-function mutations of *PTPN11* encoding SHP-2, which were mainly located in the interface between amino-terminal src-homology 2 (N-SH2) and protein-tyrosine phosphatase (PTP) domains. In contrast, NSML was associated with dominant-negative or loss-of-function mutations of SHP-2, which were located only in the PTP domain and are closed to the substrate binding or catalytic sites of SHP-2 (16, 17). The NSML associated SHP-2 mutant Tyr279Cys impaired phosphatase activity and weakened the intramolecular interaction between N-SH2 and PTP domains. The weakened interaction resulted in the N-SH2 domain being more readily activated by competing phospho-tyrosine ligands. Simultaneously, the NSML associated SHP-2 mutant Tyr279Cys increased the propensity to undergo the transition from a closed, autoinhibited conformation to an open, activated state (15, 18). The open conformation of NSML associated SHP2 mutations enhance SH2 domain protein-protein interactions that likely lead to propagating aberrant signaling and relate to the pathogenesis of NSML (15). Genotype-related phenotype heterogeneity relies on the developmental time. NSML patients may have the higher risk of hearing loss. While both NS and NSML patients develop congenital heart defects, the proportion of patients are different: the majority (85%) of NSML patients present with HCM, whereas pulmonary stenosis is predominant in NS patients (19).

As illustrated in this case report, the same *PTPN11* mutation in exon 7, c.836 A > G, p.Tyr279Cys was detected in the proband (individual II-3) and proband's son (individual III-2). Interestingly, other than HCM, the proband's son (individual III-2) presented with pulmonary stenosis and atrial septal defect, which are usually associated with NS resulting from exon 3 and 8 mutations of the *PTPN11*. To our best knowledge, these echocardiography findings have rarely been reported as the cardiac phenotypes of exon 7 mutations of the *PTPN11*. Further research on genotype and phenotype correlations is warranted.

In general, 55% of HCM cases are familial and 45% are sporadic (20). Approximately 75% of familial form of HCM is autosomal dominance inheritance (8). The natural history of HCM, particularly patterns of left ventricular remodeling may be related to clinical outcomes and risk of SCD (21). In NSML, if one parent is affected, a 50% recurrence risk is appropriate (22). Thus, complete family screening and genetic counselling is needed. Clinicians may consider whole exome sequencing study to determine the deleterious and pathogenic variants, and simultaneously to clarify any potential disease contributing NSML variation, particularly some individuals with atypical phenotypic features and proband's offspring.

## Conclusion

In summary, NSML is a rare autosomal dominant disease characterized by facial dysmorphism, specific cutaneous and cardiac manifestations. This case report highlights the clinical spectrum of this rare disease in childhood, adulthood and elderhood. As HCM-related SCD remains the leading cause of death, early and periodic cardiovascular assessments are of extreme importance for such patients. Complete family screening and genetic counselling of different generations should be performed. Additional genetic testing could shed light on genotype-phenotype correlations.

## Data Availability

The original contributions presented in the study are included in the article/**Supplementary Materials**, further inquiries can be directed to the corresponding author.
