# Supplementary material for: Case report: Distinctive cardiac features and phenotypic characteristics of Noonan syndrome with multiple lentigines among three generations in one family
Source: Front Cardiovasc Med. 2023 Aug 24;10:1225667. doi: 10.3389/fcvm.2023.1225667 (PMC10484218; doi:10.3389/fcvm.2023.1225667)
Supplement: Supplementary file 1 [file Table1.docx]

**Supplementary table 1**

Clinical characteristics and phenotypic features of the two unaffected family members (dotted-line individuals in pedigree)

|  | Proband’s nephew (individual III-1) | Proband’s aunt (individual I-7) |
| --- | --- | --- |
| Age | 12 y/o | 70 y/o |
| Sex | M | F |
| Lentigines | **-** | **-** |
| Deafness | **-** | **-** |
| Ocular hypertelorism | **-** | **-** |
| Genital anomalies | **-** | **-** |
| Short stature | **-** | **-** |
| Thick lips | **-** |  |
| Low-set ear | **-** | **-** |
| Pectus deformity (excavatum/carinatum) | **-** | **-** |
| Intellectual disability | **-** | **-** |
| Cardiac abnormalities | **-** | **-** |
